# Are pools created when restoring extracted peatlands biogeochemically similar to natural peatland pools?

**DOI:** 10.1002/eap.3052

**Published:** 2024-10-11

**Authors:** Émilie Jolin, Julien Arsenault, Julie Talbot, Mahmud Hassan, Line Rochefort

**Affiliations:** ^1^ Département de Géographie Université de Montréal Montréal Québec Canada; ^2^ Groupe de Recherche Interuniversitaire en Limnologie (GRIL) Montréal Québec Canada; ^3^ Département des Sciences biologiques Université de Montréal Montréal Québec Canada; ^4^ Department of Plant Science Université Laval Québec Québec Canada; ^5^ Peatland Ecology Research Group (PERG) Université Laval Québec Québec Canada

**Keywords:** artificial pools, biogeochemistry, dissolved organic matter, peatland open‐water pools, wetland restoration

## Abstract

In the last 25 years, several degraded peatlands in eastern Canada have been restored toward their natural structure. Pools are common in natural peatlands and are important habitats for unique flora and fauna. Because of their ecological value, pools have been created in some restored peatland sites. Nevertheless, the biogeochemistry of created pools in a restoration context has seldom been studied. The objective of our study is to characterize the biogeochemistry of created pools from restored peatlands and compare them with natural pools along a chronosequence since their creation. We measured different biogeochemical variables (pH, concentrations of nitrogen (N), phosphorus (P), dissolved organic carbon (DOC), dissolved organic matter (DOM), base cations—calcium (Ca), sodium (Na), magnesium (Mg), and potassium (K)—and dissolved gases—methane (CH_4_), carbon dioxide (CO_2_), and nitrous oxide (N_2_O)‐) in 61 pools distributed over seven peatlands in eastern Canada. The sites represent a range of conditions, from natural to restored peatlands with pools ranging from 3 to 22 years old. Created and natural pools had distinctive biogeochemistry, with created pools being generally less acidic (pH >5) and 2.5 times more concentrated in nutrients (N and P) than in natural pools. DOC, N, P, dissolved gases, and base cations concentrations were lower in natural pools than in created pools, and varied between created sites. The oldest created pools (age >17 years) tend to approach the biogeochemical characteristics of natural pools, indicating that created pools may, over time, provide habitats with similar conditions to natural pools. A return of created pools to a natural pool‐like biogeochemistry could thus inform on the success of peatland restoration.

## INTRODUCTION

Open‐water pools are common in ombrotrophic temperate and boreal peatlands, and in the coastal and maritime regions of eastern Canada (Glaser & Janssens, 1986). The biodiversity of peatlands with pools is generally higher than peatlands without pools because some species are only present in or around the pools (Desrochers & van Duinen, 2006; Poulin et al., 1999). Pools can also serve as feeding sites for amphibians and birds (Beadle et al., 2015; Mazerolle, 2005), exclusive breeding habitats for arthropods (Larson & House, 1990; van Duinen, 2013), and resting areas for migratory birds (Desrochers, 2001). Hence, pools contribute greatly to the overall biodiversity of the peatlands where they are present.

Peatland pools are shallow (<2 m) and their surface area is generally comprised between 0.1 and 10,000 m^2^ (Arsenault et al., 2022). The depth of natural pools is primarily limited by the thickness of peat deposits in which they develop (Belyea & Lancaster, 2002; Foster & Fritz, 1987). Shallow (<1 m) pools are especially dynamic and emit more carbon (C) than deeper pools (McEnroe et al., 2009). Pools can often be net C sources to the atmosphere (McEnroe et al., 2009), although C sequestration in undisturbed pool‐rich peatlands generally exceeds C emission from pools (Pelletier et al., 2014). Along with C, biogeochemical patterns and processes of N and P in pools are also controlled by depth and the composition of the surrounding vegetation. For example, shallow pools and those surrounded by high proportions of ligneous vegetation tend to have higher concentrations in total N and P (Arsenault et al., 2018, 2019). Depth influences light penetration, water temperature, and oxygen concentrations at the bottom of the pools, stimulating the biological activity of production and decomposition at the water–sediment interface (Hamilton et al., 1994; Karofeld & Tõnisson, 2014).

Despite providing several critical ecosystem services (Kimmel & Mander, 2010), about 10% of the world's peatlands are disturbed or destroyed by anthropogenic activities (UNEP, 2022). In the last 25 years, peatland restoration techniques have been developed and successfully applied (Chimner et al., 2017), allowing, in some cases, the recovery of their C sink function (Glenk & Martin‐Ortega, 2018; Nugent et al., 2018). In North America, restoration efforts focus on the reestablishment of typical peatland vegetation and suitable hydrological conditions to facilitate a rapid return of the accumulation of plant biomass (e.g., Gorham & Rochefort, 2003). In many restoration projects, pools spontaneously appeared after drainage ditch blocking (e.g., Parry et al., 2014), or were mechanically created to regenerate the natural landscape of peatlands (e.g., Mazerolle et al., 2006). Generally, water level fluctuates more in created than in natural pools (Holden et al., 2018), and DOC and dissolved CO_2_ are more concentrated in created pools (Chapman et al., 2022). It is, however, not clear how mechanically created pool biogeochemistry evolves over the long term (>20 years). In this context, our objective was to evaluate the success of pool creation during peatland restoration using common biogeochemical indicators by (1) identifying the biogeochemical differences between created and natural pools, and (2) assessing the biogeochemical trajectory of pools that have been mechanically created between 3 and 22 years ago.

## METHODOLOGY

### Sites description

**FIGURE 1:**
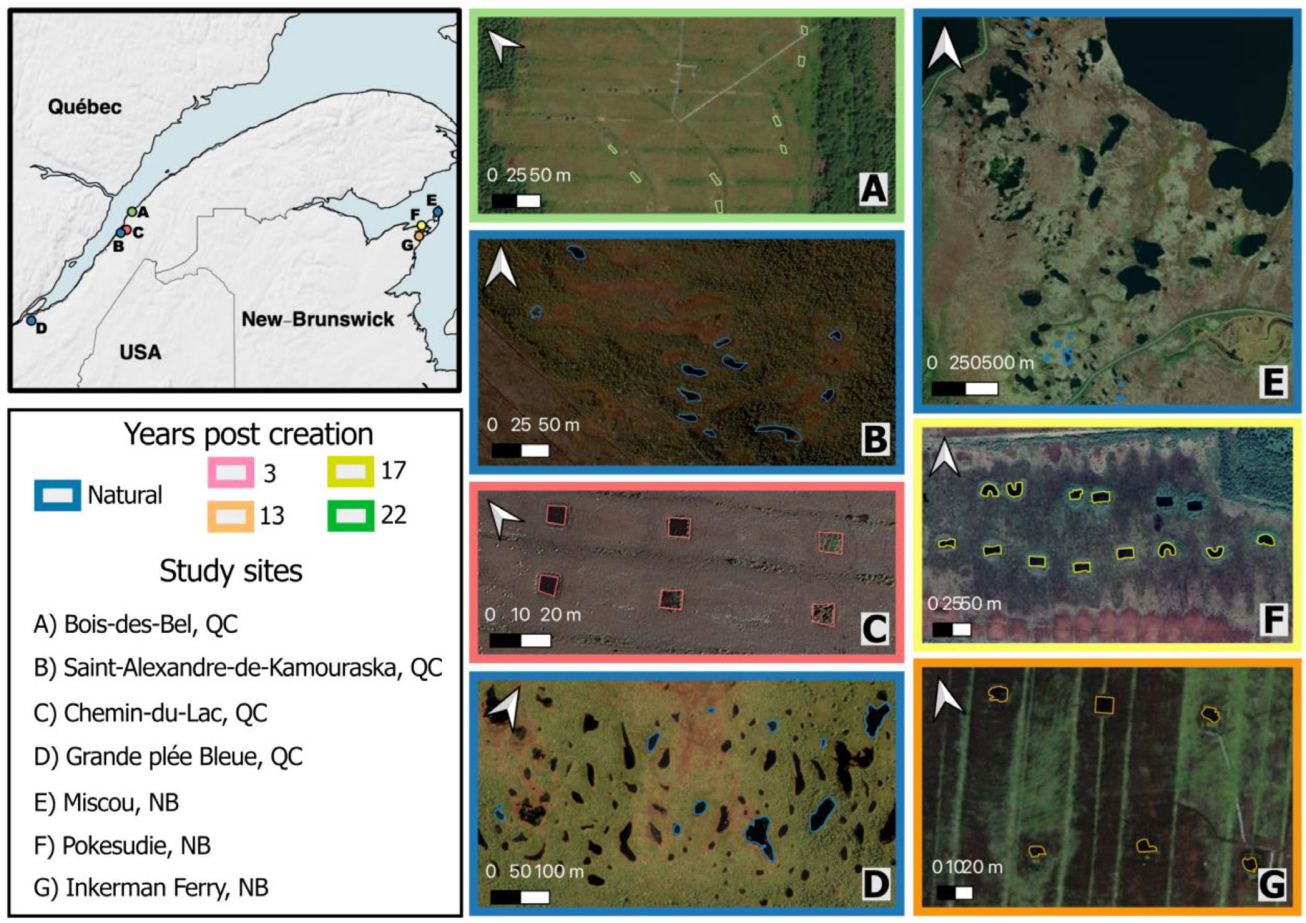
Location of the study sites in Quebec and New Brunswick. Maps based on Google Maps satellite images, 2023.

We conducted this study in seven peatlands distributed in Québec and New Brunswick, in eastern Canada (Figure 1). Four of the sites were located in Québec in the St. Lawrence lowlands, and the other three sites were located in New Brunswick in the Acadian peninsula. Three of the study sites were unexploited bogs with natural pools (two in Québec and one in New Brunswick), and the other sites were bogs with pools mechanically created during peatland restoration (two in Québec and two in New Brunswick). Peatlands of the Acadian peninsula formed around 11,000 BP (Rampton et al., 1984), while bogs in Québec have developed following the withdrawal of the Goldthwait Sea (Dionne, 2010), around 9500 BP for the site near Québec City (M. Lavoie et al., 2012), and 8000 BP for the other sites (Richard et al., 1992).

In natural sites, vegetation was dominated by *Sphagnum* mosses (e.g., *Sphagnum fuscum*, *S. medium*, and *S. angustifolium*) with a cover of ericaceous shrubs (*Chamaedaphne calyculata*, and *Kalmia angustifolia*), graminoids (e.g., *Rhynchospora alba* and *Eriophorum vaginatum*), and trees (*Picea mariana* and *Larix laricina*), although tree coverage varies from very sparse at Miscou in New Brunswick to dense in Saint‐Alexandre‐de‐Kamouraska in Québec. Surface conditions were dryer at the more maritime Miscou peatland, where lichen and *Gaylussacia baccata* are common.

Natural pools at Grand plée Bleue (*n* = 9) and Miscou (*n* = 9) were selected to represent a large variety of pool morphology: small and shallow, small and deep, and large and shallow pools. At Saint‐Alexandre‐de‐Kamouraska (*n* = 11) all naturally occurring pools were sampled. We also studied created pools in four peatlands that have been peat extracted for growing media and restored from 3 to 22 years ago (Figure 1): Bois‐des‐Bel (*n* = 8; 22 years), Pokesudie (*n* = 12; 17 years), Inkerman Ferry (*n* = 6; 13 years), and Chemin‐du‐Lac (*n* = 6; 3 years) (Table 1). In each restored peatland, pools were created following the method described in Quinty and Rochefort (2003).

**TABLE 1:** Summary characteristics of natural and restored sites and pools.

Pool type and study site	Latitude; longitude	Total annual precipitation (mm)	Mean annual air temp. (°C)	Years post‐creation	No. pools	No. sampling events	Mean depth (cm)	Mean area (m^2^)	Mean underlying peat (cm)
Created pools									
(A) Bois des Bel, QC^a^	47,967; −69,429	963.5	3.5	22	8	8	17 (±3)	49 (±12)	126 (±11)
(F) Pokesudie, NB^b^	47,819; −64,778	1077.2	4.8	17	12	1	55 (±16)	237 (±39)	125 (±16)
(G) Inkerman Ferry, NB^b^	47,705; −64,818	1077.2	4.8	13	6	1	32 (±14)	59 (±18)	369 (±14)
(C) Chemin du Lac, QC^a^	47,773; −69,515	963.5	3.5	3	6	8	24 (±2)	40 (±6)	76 (±2)
Natural pools									
(D) Grande plée Bleue, QC^c^	46,782; −71,046	1178.7	4.6	…	9	8	125 (±57)	642 (±629)	366 (±61)
(E) Miscou, NB^b^	47,966; −64,516	1077.2	4.8	…	9	1	86 (±57)	889 (±1347)	414 (±57)
(B) St‐Alexandre‐de‐Kamouraska, QC^a^	47,740; −69,610	963.5	3.5	…	11	8	67 (±18)	93 (±59)	433 (±18)

*Note*: Climate data is based on Canadian Climate Normal 1981–2010 retrieved from the closest meteorological stations. Underlying peat is calculated based on total peat depth of each site minus average pool depth.

^a^ Saint‐Arsène meteorological station no. 7056890.

^b^ Haut Shippagan station no. 8102206.

^c^ Lauzon station no. 7024254.

At the Bois‐des‐Bel site, peat extraction ended in 1980 (C. Lavoie et al., 2001) leaving behind a layer of peat up to 2 m thick over a clay substrate. The restoration took place in 2000 using the moss layer transfer technique, and vegetation has successfully reestablished a *Sphagnum* mosses‐dominated carpet (Rochefort et al., 2013). Specific peatland plants such as *C. calyculata*, *Rhododendron groenlandicum*, and *E. vaginatum* have reestablished, but some non‐bog species have also colonized the site such as *Typha latifolia* which grows in and around the pools. Eight pools were created with initial dimension of 6.5 m × 12 m (78 m^2^) with a rectangular shape, and depth varying between 1 and 1.5 m (22 years ago; Rochefort & Lode, 2006). The pools had a steep slope on one side and a gentle slope on the opposite side to represent the natural variability of pool topography (Quinty & Rochefort, 2003).

At the Pokesudie site, peat extraction ended in 2001. The site was restored in 2004 using the moss layer transfer technique (17 years ago; Fontaine, 2008). Vegetation has re‐colonized the site and is mostly composed of graminoids (*Eriophorum angustifolium*, *Juncus* spp. and *Carex* spp.), shrubs (*Myrica gale*, *Kalmia angustifolia*, and *Vaccinium macrocarpon*) and peat mosses (*Sphagnum* spp. and *Polytrichum* spp.). Fourteen pools were created (12 were sampled), nine with a rectangular shape (8 m × 22 m) and five with a U‐shape (8 m × 25 m), all with a shallow and a steep bank (Fontaine, 2008). The initial depth varied between 0.5 and 1 m. When the pools were created, mineral soil (mostly sand) was reached due to the shallow residual peat (Fontaine, 2008).

At the Inkerman‐Ferry site peat extraction ended in the early 1990s, leaving a layer of peat up to 4 m thick (Laberge et al., 2013), with a clay base substrate. The site was restored in 2008, thus the pools had been created 13 years ago at sampling time. Sparse vegetation composed mainly of graminoids (*Eriophorum* spp. and *Carex* spp.), shrubs (*K. angustifolia*, *K. polifolia*, *R. canadense*, *R. groenlandicum*, and *Vaccinium* spp.), and moss (*Sphagnum* spp. and *Polytrichum* spp.) had colonized the pool surroundings following restoration. Six rectangular pools were created, all with areas of 130 m^2^, depths up to 1.5 m, and shallow banks (Laberge et al., 2013).

At the Chemin‐du‐Lac site (Table 1), peat extraction ended before 2000, leaving behind a layer of peat up to 1 m thick over clay deposits. As of 2021, vegetation restoration has not yet been implemented but some plants have appeared spontaneously such as *Polytrichum* spp., *K. angustifolia*, *R. alba*, and *Eriophorum* spp. Also, three of the six pools had *T. latifolia* growing inside and around pools. Six pools were created in 2018 (3 years of age at sampling time), and the initial dimension of each pool was 8 m × 8 m (64 m^2^). Pools were square‐shaped, gently sloping on every side, and the initial depth varied from 0.7 to 1 m.

### Pool measurements and sampling

We sampled 61 pools in total: 29 natural and 32 created. In the Québec natural (*n* = 20) and restored (*n* = 14) sites, we sampled pool water four times during each of the 2020 and 2021 growing seasons (from May to September). We also sampled natural (*n* = 9) and created (*n* = 18) pools from New Brunswick one time in July 2021. We determined the average depth of each pool by dropping a Secchi disk (400 g, 20 cm diameter) to their bottom at different points in the pool (3–10 times depending on its size). We measured pool area based on the presence of visible open water using 0.46 m resolution satellite imagery. We described the vegetation surrounding each pool by evaluating cover percentage for each stratum (moss, grass, shrub, and tree).

In each pool, we took water samples 20 cm below the surface at a 2 m distance from the edge of the pool using a pole. If the pool was less than 20 cm deep, water was collected from the surface. We collected water samples in polypropylene tubes for pH, total nitrogen (TN) and phosphorus (TP), nitrate (NO_3_), ammonium (NH_4_), phosphate (PO_4_), base cations (calcium—Ca, sodium—Na, magnesium—Mg, and potassium—K) analyses. DOC and dissolved organic matter (DOM) samples were collected in 50 mL pre‐acid washed amber borosilicate vials after removing particulate matter through 0.45 μm polyethersulfone (PES) membrane syringe filters. The NO_3_, NH_4_, and PO_4_ samples were filtered using a 0.2 μm PES membrane syringe filter. The NO_3−_ and NH_4_^+^ samples were acidified in the field at pH <2 using 0.1 M sulfuric acid (H_2_SO_4_). The rest of the water samples were unfiltered. Dissolved CO_2_, CH_4_ and N_2_O samples were also collected at the pool surface using the headspace sorptive extraction technique (e.g., Kling et al., 1991). All water samples collected during the field campaigns were kept at 4°C until analysis, except for PO_4_ samples, which were kept at −20°C.

### Laboratory analyses

The laboratory chemical analyses we used followed the approach described in Arsenault et al. (2018). We measured water pH using an Accumet AB150 pH meter (Fisher Scientific, USA) shortly after sampling. We used colorimetric analyses after chemical digestions for TP and TN. Digestions used a potassium persulfate and oxidizing reagent (a solution of NaOH and potassium persulfate), for TN and TP, respectively. Total N, NO_3_, and NH_4_ analyses were performed on a Lachat QuikChem 8000 flow injection autoanalyzer (Lachat Instruments, USA). Total P and PO_4_ analyses were performed on an Astoria 2 segmented flow analyzer (Astoria‐Pacific, USA). Dissolved gas analyses were carried out using a Shimadzu GC‐2014 gas chromatograph (Shimadzu Scientific Instruments, USA). DOC concentration was measured using the wet oxidation method with an Aurora 1030 TIC‐TOC Analyzer (IO Analytical Instruments, USA) by calculating the difference between total carbon (TC) and inorganic carbon (IC). Base cations concentrations were measured using a 55‐AA atomic absorption spectrometer (Agilent, USA). We also measured UV–visible absorbance between 190 and 900 nm using a Shimadzu UV‐1800 spectrophotometer (Shimadzu Scientific Instruments, USA) using a 10 mm‐path quartz cuvette. We then calculated the specific UV absorbance (SUVA) by dividing the UV absorbance at 254 nm (cm^−1^) by the DOC concentration (mg L^−1^), and used it as a proxy for DOM aromaticity (Weishaar et al., 2003). DOM fluorescence spectra were scanned using a Varian Cary Eclipse Fluorescence Spectrophotometer (Agilent Technologies, USA) with a 10 mm‐path quartz cuvette at room temperature by measuring fluorescence intensity across 220–450 nm (5‐nm increments) excitation wavelengths, and 230–600 nm (2‐nm increments) emission wavelengths at 5 nm slit widths.

### Data treatment and statistical analyses

We first described the relationships between depth and water chemistry (pH, DOC, TN, TP) from natural and created pools with simple regression analyses using only data from the July 2021 samplings (*n* = 61; 30 natural and 32 created), when all sites were sampled. We distinguished created pools from others using years post‐creation as descriptors. To determine whether pool type (natural vs. created) had any effect on pool characteristics and biogeochemistry, we ran generalized linear models (GLM) on physical (depth and area) and biogeochemical variables (DOC, TN, TP, NH_4_, NO_3_, PO_4_, Ca, Mg, Na, K, and dissolved CO_2_, CH_4_, and N_2_O concentrations, as well as pH and SUVA). For all variables, we used gamma distribution families. Then, to explore the effect of time since creation on created pool biogeochemistry, we ran other GLMs based on the same subset of pools sampled in July 2021 as previously described. For each variable, we ran the model twice, once with natural pools as the reference system and a second time with the 3 years post‐creation pools as the reference to determine how pools of different age compared with old, natural, and newly created pools. We also ran generalized linear mixed models on all pools, regardless of sampling time, to determine the spatiality of both natural and created pool biogeochemistry. We used gamma distribution families for all biogeochemical properties, with pool type as fixed effects, and added sites and regions as random effects.

To evaluate the temporal evolution of natural and created pool biogeochemistry, we first performed a principal components analysis (PCA) using only variables for which we gathered data over the 2020 and 2021 growing seasons (pH, DOC, NH_4_, NO_3_, TN, TP, PO_4_, CO_2_, CH_4_, N_2_O and SUVA). We compared created pools (*n* = 14) with those from the natural sites, (*n* = 20) and distinguished created pools based on their age (3‐ or 22‐years post‐creation). We then ran generalized linear mixed models to determine how pool age influenced pH and DOC, TN, and TP concentrations regardless of temporality and pool size. We used gamma distribution families for all biogeochemical properties and added sampling time and pool physical characteristics as random effects.

To determine the drivers of biogeochemical changes over time, we performed a redundancy analysis (RDA) using data from the 61 pools surveyed in July 2021 at all seven sites. We used environmental variables (pool depth, area, underlying peat, and strata vegetation) as predictors and biogeochemical variables (pH, DOC, NH_4_, NO_3_, TN, TP, PO_4_, K, Mg, Na, Ca, CO_2_, CH_4_, N_2_O, and SUVA) as responses. Again, we compared created pools (*n* = 32) with those from the natural sites (*n* = 29) and distinguished created pools based on their age (3‐, 13‐, 17‐, and 22‐years post‐creation). The best environmental variables were selected using a forward selection, and we standardized the response matrix. The significance of the RDA axes was tested by permutations. Vegetation cover percentages were transformed using the Hellinger transformation and the normality of the distribution of physicochemical variables was tested using Shapiro–Wilk tests. Non‐normally distributed variables were log‐transformed to fulfill normality assumptions. All statistical analyses were performed with R, version 4.4.1 (R Core Team, 2022).

Finally, to get a better understanding of the composition of DOM, we characterized and identified DOM components differences and similarities between created and natural pools. For that, we used DOM fluorescence spectra that were corrected for instrument bias, inner filter effect and Raman scattering prior to parallel factor analysis (PARAFAC) in Matlab using the script from LaBrie et al. (2017). Corrected fluorescence spectra were used to conduct PARAFAC analyses which decomposes the excitation and emission matrices by grouping fluorescent compounds with a similar molecular structure into different components (Stedmon et al., 2003) using MATLAB with drEEM toolbox (Murphy et al., 2013). The online repository OpenFluor database was used for the comparison of the PARAFAC components with published studies (Appendix S1: Table S5) (Murphy et al., 2014).

## RESULTS

### Morphology of created and natural pools

At our sites, natural pools were deeper than created pools (Table 2; Appendix S1: Table S1). Natural pool depth varies between 219 and 32 cm (mean = 93 cm), while created pool depth varies between 86 and 12 cm (mean = 25 cm). Pool area was generally similar among restored sites, except for the 17 years post‐creation site where pools were larger (237 m^2^ on average) and similar in size to natural pools (Appendix S1: Table S2). Over time, water depth in created pools was more variable than in natural pools. For instance, created pools at the BDB (22 years post‐creation) and CDL (3 years post‐creation) sites had sometimes completely dried up (Table 3), but natural pools only had very little water level fluctuation (10–20 cm) within and among the two field seasons.

**TABLE 2:** Means (±SD) of physical and chemical properties of natural and created pools from the Québec and New Brunswick sites sampled in July 2021.

Pool properties	Natural (*n* = 30)	22 years (*n* = 8)	17 years (*n* = 12)	13 years (*n* = 6)	3 years (*n* = 6)
Depth (cm)	90 (±52)	22 (±12)	55 (±16)	32 (±14)	21 (±6)
Area (m^2^)	508 (±864)	49 (±12)	237 (±39)	59 (±18)	40 (±6)
Underlying peat (cm)	410 (±52)	128 (±12)	125 (±16)	369 (±14)	79 (±6)
pH	4.1 (±0.2)	4.9 (±0.2)	4.4 (±0.5)	5.2 (±0.7)	5.5 (±0.2)
DOC (mg L^− 1^)	29.7 (±9.5)	99.38 (±23.9)	25.1 (±12.1)	61.7 (±19.5)	154.0 (±48.5)
TN (mg L^− 1^)	1.0 (±0.49)	2.0 (±0.2)	0.8 (±0.2)	1.0 (±0.2)	8.0 (±2.7)
TP (μg L^−1^)	21.4 (±199)	41.9 (±9.7)	46.8 (±31.1)	53.9 (±61.1)	84.4 (±74.1)
NH_4_ (μg L^−1^)	28.4 (±18.0)	94 (±39.1)	68.8 (±65.4)	84.7 (±24.5)	1578.9 (±1820.6)
NO_3_ (μg L^−1^)	8.0 (±4.3)	55.8 (±32.1)	8.9 (±5.8)	22.7 (±4.9)	121.3 (±49.0)
PO_4_ (μg L^−1^)	3.2 (±1.7)	8.2 (±2.9)	7.6 (±5.2)	26.1 (±37.4)	20.4 (±9.5)
SUVA (L mg C^−1^ m^−1^)	4.04 (±1.02)	4.69 (±0.64)	4.15 (±0.95)	4.38 (±0.48)	5.08 (±0.19)
CH_4_‐C (μg L^−1^)	73.3 (±76.3)	42.2 (±65.3)	22.7 (±23.6)	181.1 (±204.8)	3.7 (±3.4)
CO_2_‐C (μg L^−1^)	1112 (±880)	1735 (±1835)	697 (±546)	2917 (±4003)	1277 (±683)
N_2_O (μg L^−1^)	3.0 (±3.1)	1.4 (±0.1)	11.2 (±6.9)	26.9 (±33.1)	0.9 (±0.1)
Ca (mg L^−1^)	0.17 (±0.07)	3.24 (±1.10)	0.66 (±0.68)	0.60 (±0.70)	1.75 (±0.69)
Mg (mg L^−1^)	0.29 (±0.2)	4.97 (±0.94)	1.77 (±0.57)	1.56 (±0.85)	2.12 (±0.38)
K (mg L^−1^)	0.38 (±0.25)	1.21 (±0.35)	0.95 (±0.38)	1.05 (±0.63)	4.04 (±2.22)
Na (mg L^−1^)	1.86 (±1.67)	32.44 (±10.05)	11.17 (±2.10)	4.21 (±1.36)	16.39 (±4.23)

Abbreviations: DOC, dissolved organic carbon; TN, total nitrogen; TP, total phosphorus.

**TABLE 3:** Percentage of pools at the Québec restored sites that were dried up at the time of sampling during the 2020 and 2021 growing seasons.

Time of sampling	22 years (*n* = 8)		3 years (*n* = 6)	
	Pools dried up (*n*)	Dried up pool percentage (%)	Pools dried up (*n*)	Dried up pool percentage (%)
June 2020	2	25	2	33
July 2020	3	38	3	50
August 2020	5	63	6	100
September 2020	3	38	2	33
May 2021	0	0	0	0
June 2021	0	0	0	0
July 2021	0	0	0	0
August 2021	1	13	2	33

### Water chemistry

Created and natural pools had distinctive biogeochemistry (Appendix S1: Table S1). Pools were less acidic in created pools (mean across ages pH = 5.0) than in natural pools (mean across natural sites pH = 4.1) and followed a general gradient of higher pH in younger sites (<13 years old) (Table 2; Appendix S1: Table S2). DOC concentrations in created pools (mean = 99.4 mg L^−1^) were generally higher than in natural pools (mean = 32.1 mg L^−1^) but were the lowest of all created and natural sites at the 17 years post‐creation site (mean = 30.2 mg L^−1^). Similarly, N, P, dissolved gases, and base cations concentrations were lower in natural pools than in created pools, and varied between created sites (Table 2; Appendix S1: Tables S2 and S3) but not so much among regions (Appendix S1: Table S3). Data from the repeated samplings in the Québec sites also showed that the younger created pools had, for the most part, higher nutrients (TN, TP, NH_4_, NO_3_, PO_4_, Ca, Mg, K, Na) and dissolved gas concentrations than natural pools (Table 2).

**FIGURE 2:**
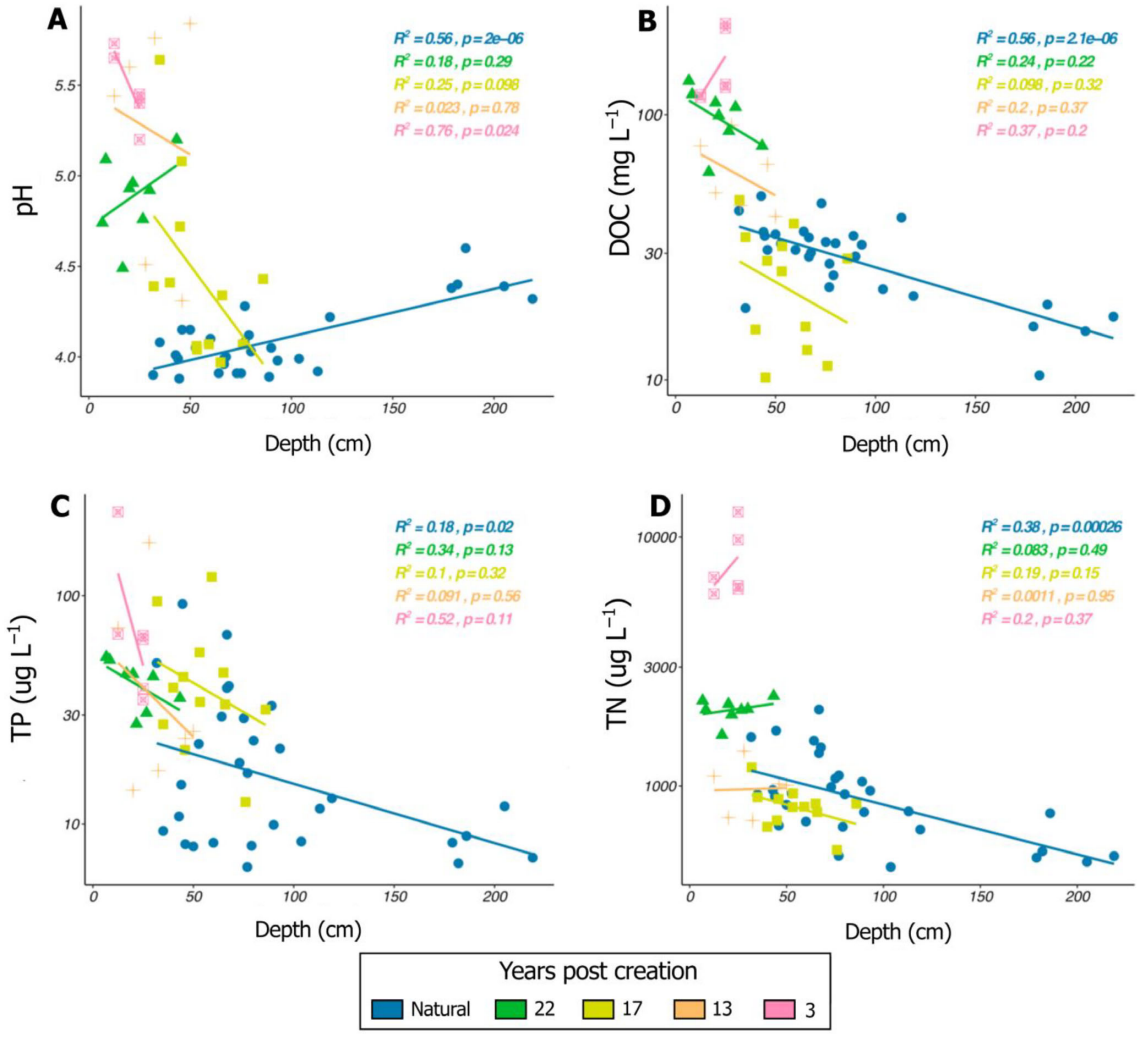
Regressions between pool depth and pH, dissolved organic carbon (DOC), total phosphorus (TP), and total nitrogen (TN) in natural and created pools of different ages. Colors and symbols represent different pool types and age (natural or created 3, 13, 17, or 22 years ago).

Natural pools showed patterns of increasing pH with increasing depth while created pools showed decreasing pH with increasing depth (Figure 2). Patterns of decreasing DOC concentrations with increasing pool depth were however similar between natural and created sites, but created pools generally had much higher DOC concentrations, reaching up to three times the concentration measured in natural pools of the same depth (Figure 2). Although TN and TP concentrations were up to two orders of magnitude higher in created than in natural pools, the relationships of TN and TP concentrations to depth were not significant in created pools (*p* < 0.1), contrary to natural pools in which TN (*p* = 0.02) and TP (*p* < 0.001) concentrations decreased with increasing pool depth.

### Dissolved organic matter composition

**FIGURE 3:**
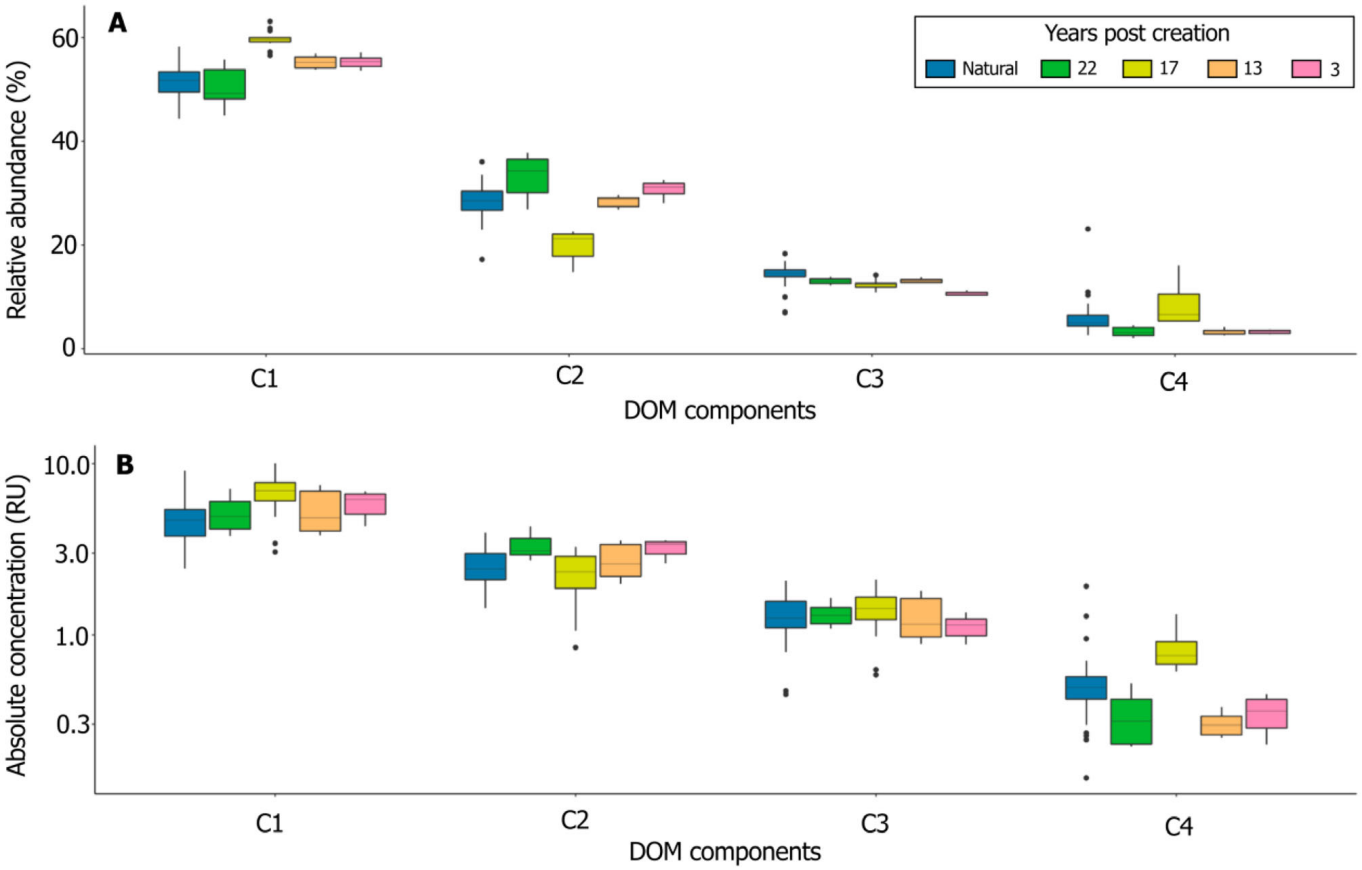
Dissolved organic matter (DOM) components of all sites in July 2021. (A) Boxplots of absolute concentration. (B) Boxplots of relative abundance. The 4‐component PARAFAC model explains 99.85% of the total variation, where C1 represents terrestrially derived humic‐like DOM with high molecular weight degraded from lignin; C2 represents terrestrial humic‐like DOM, C3 represents terrestrial humic‐like, high relative aromaticity and molecular weight, and C4 represent protein‐like (mixture of tyrosine‐type and tryptophane‐type compounds), freshly produced DOM. Colors represent different pool types and ages (natural or created 3, 13, 17, or 22 years ago).

We validated a 4‐component PARAFAC model that explained 99.8% of the variation among DOM fluorescence data. The four components (C1–C4) showed the same patterns for both the relative abundance and the absolute concentration in DOM components (Figure 3). For example, component C1, representing terrestrially derived humic‐like DOM with high molecular weight degraded from lignin, and component C2, also representing terrestrial humic‐like DOM (Hassan et al., 2023; Wünsch et al., 2017; Yamashita et al., 2010), were generally higher in the created than in the natural pools. Opposingly, components C3 (terrestrial humic‐like with high relative aromaticity and molecular weight) (e.g., Wünsch et al., 2017) and C4 (protein‐like, tyrosine‐type and tryptophane‐type compounds) (e.g., Yamashita et al., 2010), showed higher proportion and abundance of freshly produced DOM in natural than in created pools. Especially for C1, which was the most abundant DOM component in pools of all sites, DOM composition in older created pools tended to resemble that of natural pools.

### Patterns and drivers of pool biogeochemical temporal evolution

**FIGURE 4:**
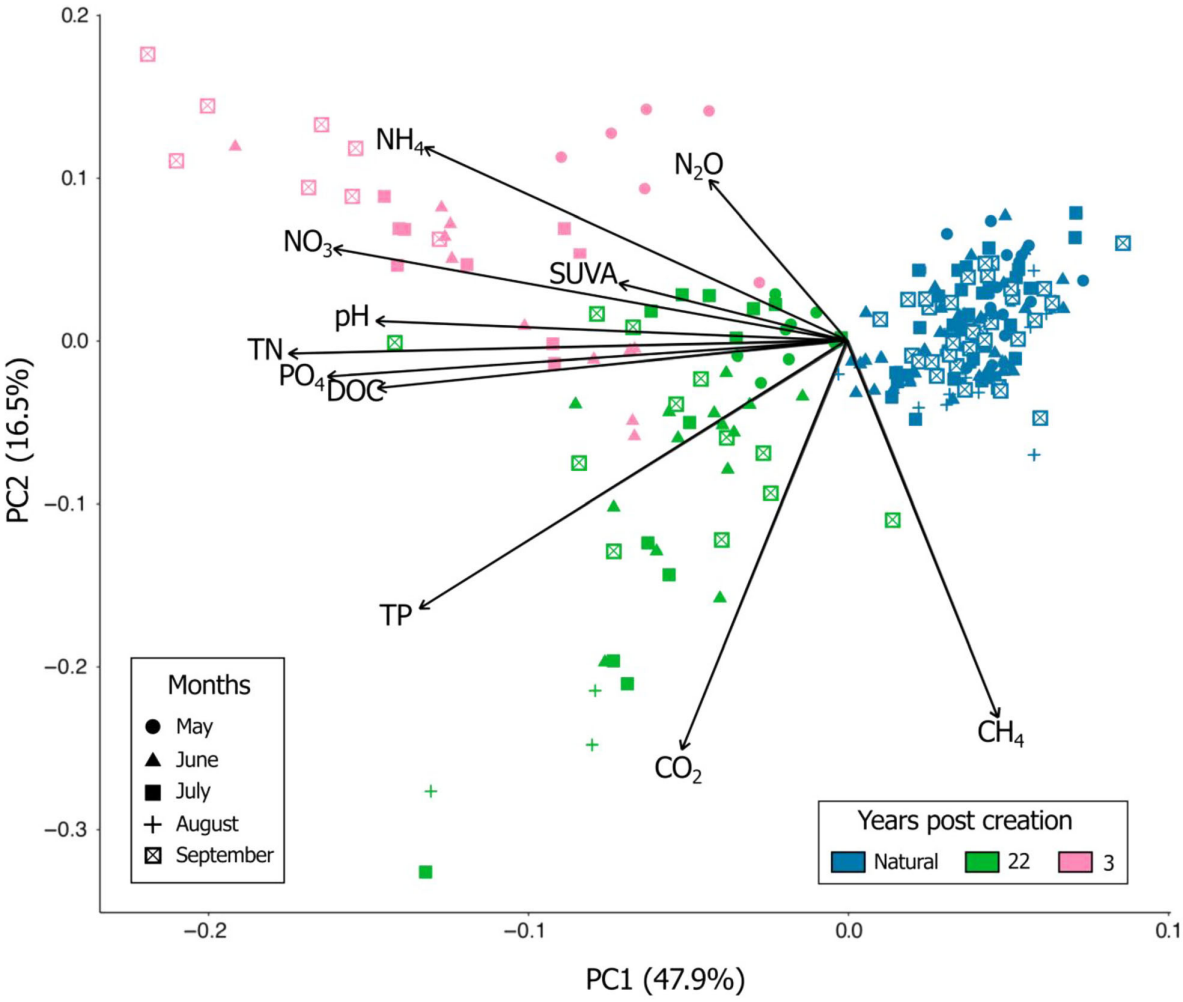
Principal component analysis of natural (*n* = 160) and created (4 years post‐creation, *n* = 48; 22 years post‐creation, *n* = 64) pool water chemistry from the 2020 and 2021 growing seasons. Pool biogeochemistry is mostly driven by pool type, while seasonality only had a limited effect in the differentiation of created and natural pools. Colors represent different pool types and ages (natural or created 3, 13, 17, or 22 years ago) and symbols represent sampling periods during the growing season.

There were clear distinctions between pools of different types and ages from the Québec sites, as illustrated by the PCA (Figure 4). There was however only a small discernible effect of sampling time among pool types, with temporal pattern emerging only in the 3 years post‐creation pools. These pools had higher concentrations in C, N, and P at the end compared with the beginning of the growing seasons. The large range of pool distribution in the PCA showed that temporal variations in biogeochemistry over the 2020 and 2021 growing seasons were more pronounced in the created than in the natural pools (Figure 4). Generalized linear mixed models also showed strong differences in pool biogeochemistry between the natural, the 22 years post‐creation, and the 3 years post‐creation pools (Appendix S1: Tables S4 and S6), but variable influence of sampling time and pool physical characteristics. For example, while DOC and TP concentrations were strongly driven by seasonality and pH was more influenced by pool attributes, these parameters had very little random effect on TN concentrations.

**FIGURE 5:**
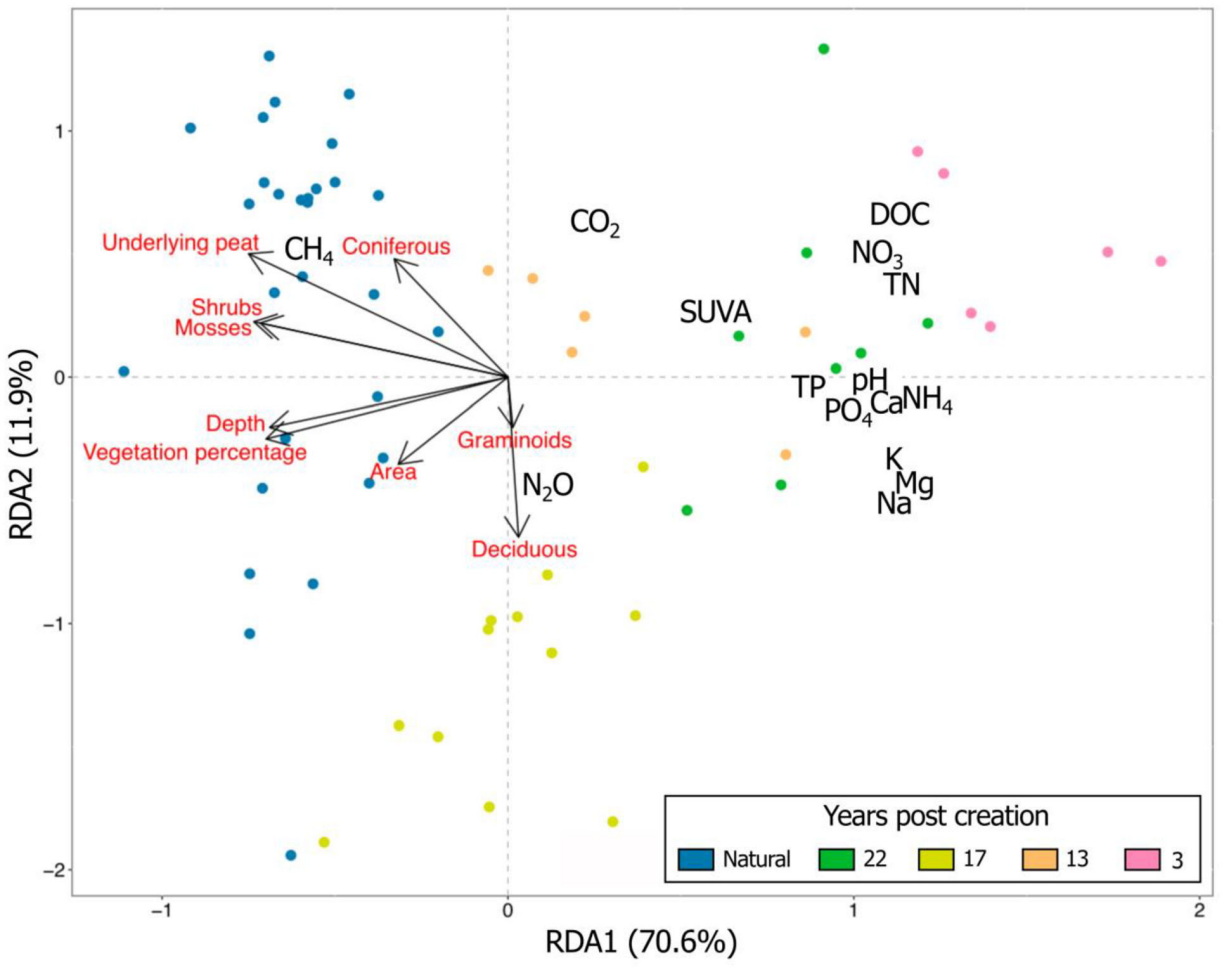
Type‐two redundancy analysis showing the influence of pool morphology and surrounding vegetation on the water chemistry of 61 pools (29 natural and 32 created) sampled in July 2021. Colors represent different pool types and age (natural or created 3, 13, 17, or 22 years ago).

Patterns also emerged when comparing the biogeochemistry and environmental characteristics of pools from all sites (Figure 5). Alongside differences in pool size, natural pools had more vegetated surroundings and had thicker layers of underlying peat than created pools. To a lesser extent, the 17‐years‐old site was the most vegetated of all restored sites. The RDA we performed showed that these characteristics explained 56.8% of the variability in pool biogeochemistry. Pools with higher proportions of mosses, shrubs, and coniferous trees tended to have higher concentration in dissolved CH_4_, but lower nutrient concentrations than created pools. The RDA also confirmed that nutrient concentrations tended to be higher in younger than in older created pools.

## DISCUSSION AND CONCLUSION

### Created and natural pools are biogeochemically different

**FIGURE 6:**
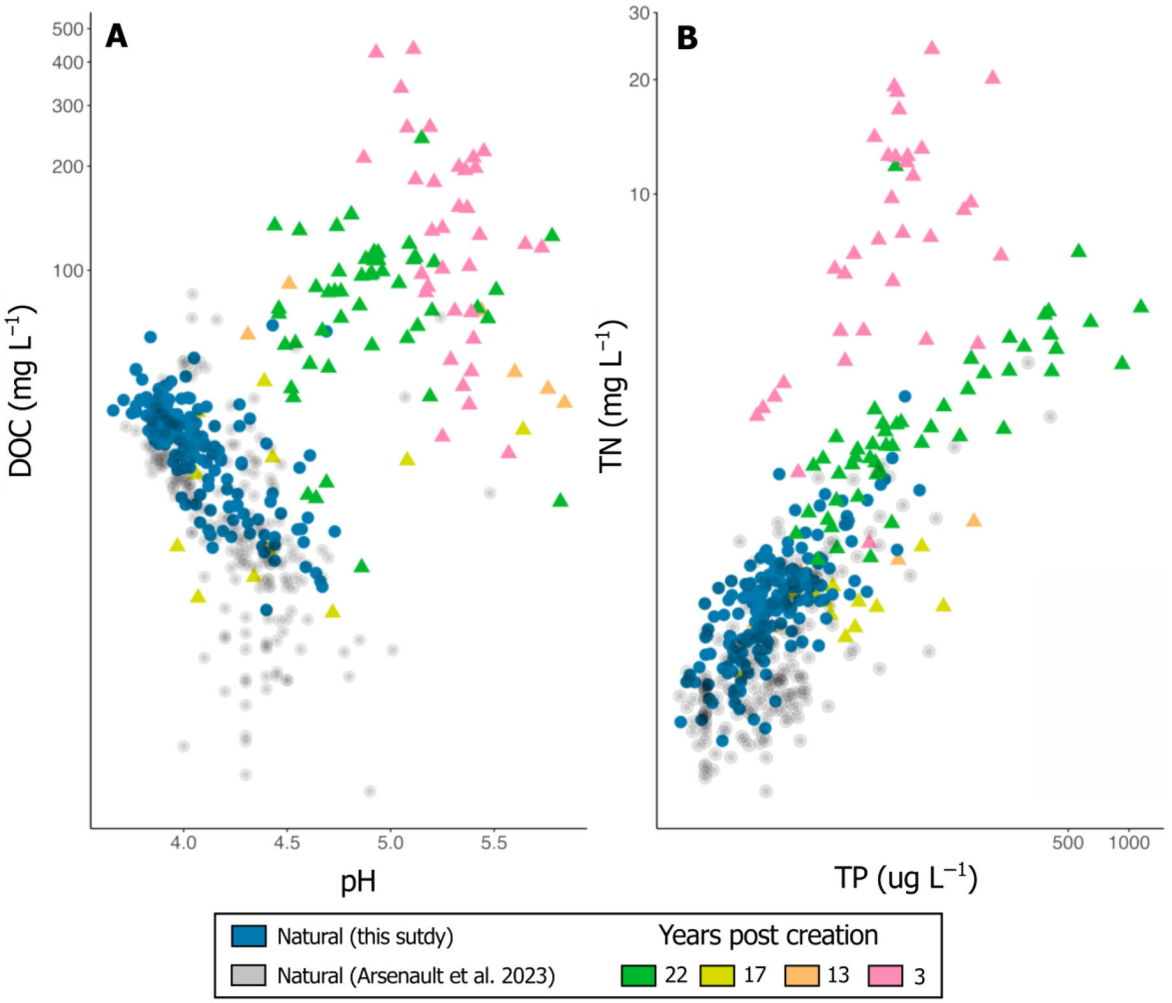
Comparison of pH, and dissolved organic carbon (DOC), total nitrogen (TN) and total phosphorus (TP) concentrations from natural pools of Europe, North America, and Patagonia (*n* = 240, in gray; taken from Arsenault et al., 2023), and those we gathered at our study sites in 2020 and 2021. From our study, natural pools (*n* = 173) and created pools (*n* = 102) of all study sites and sampling periods are presented. Colors and symbols represent different pool types and age (natural or created 3, 13, 17, or 22 years ago).

The created pools that we surveyed in eastern Canada were structurally and biogeochemically different from natural pools. However, the biogeochemistry of older pools tends to resemble that of natural pools. While the pH, DOC, TN, and TP concentrations of natural pools in this region were similar to other widely distributed natural peatland pools (Figure 6), the biogeochemistry of created pools was mostly out of range for the same variables. For example, when comparing our data with the global dataset from Arsenault et al. (2023), DOC, TN, and TP concentrations in pools from our restored sites were up to several orders of magnitude higher than in natural pools.

Created pools had variable surface area and depth among and within sites, and area and depth were smaller in restored than in natural sites. However, such variations in pool size could not explain the large range of DOC and nutrients concentrations we observed in our study. Indeed, pH, DOC, TN, and TP concentrations in created pools were several times higher than in natural pools regardless of their geographical settings and morphometry (Figure 6). The strong relationships between pool depth and biogeochemistry that exist in natural pools were also not observed in our created pools (Figure 2), meaning that environmental factors other than pool size control the spatial and temporal variability in the biogeochemistry of mechanically created pools in restored peatlands.

In ombrotrophic peatlands, water acidity mainly depends on both peat chemistry and vegetation composition, with both factors controlling the production and dissolution of organic acids (Rydin & Jeglum, 2013). The peatlands we studied were located in similar geographical settings, hence we would not expect large differences in peat chemistry among sites (Gorham & Janssens, 2005), although created pools are located in older peat that may be chemically different from the younger peat of natural sites. Additionally, peatland pool pH has been related to the composition of the vegetation surrounding the pools, with pH decreasing when the proportion of coniferous versus shrubs and mosses coverage increase (Arsenault et al., 2018; Arsenault et al., 2019), as we found in our pools (Figure 5). Unfortunately, we did not analyze the chemistry of the peat surrounding the pools, which could have further indicated a potential influence of soils on pool water. However, based on the literature, we can assume that peat chemistry indeed affects pool water chemistry by controlling the composition of the soil solution that reaches the pools during and after precipitation events (Arsenault et al., 2019; Kaštovská et al., 2018).

Our data showed that for pools of the same depth, DOC concentration was generally higher in created than in natural pools. Previous studies have shown that pool DOC concentration increased with decreasing pool depth, in both natural (Arsenault et al., 2018) and artificial pools (Chapman et al., 2022). Regardless of pool depth, high DOC concentrations in ombrotrophic peatland pore water have previously been related to water table fluctuations (Strack et al., 2008). This could partly explain the differences we observed: pool and water table depth were much more variable in restored than in the natural sites, as shown in Holden et al. (2018). In created pools, we also often measured very small water volumes (Tables 2 and 3), but these measurements varied over time in relation to weather conditions. Similar processes related to water level fluctuations could explain the higher N, P, and base cation concentrations measured in created pools. Higher decomposition rates in surrounding peat caused by soil aeration and warmer water temperature during low water table episodes would indeed mobilize nutrients that would not be already consumed by plants due to very low vegetation covers in restored sites (Wind‐Mulder et al., 1996). It is then possible that water level fluctuations in pools and the surrounding peat in relation to alternating dry and wet periods led to cycles of high nutrient production and mobilization in created pools.

Dean et al. (2023) showed that both DOC and dissolved CO_2_ in peatland pools were mostly composed of contemporary (<300 years) C, and that the proportion of relatively young C was higher in artificial than in natural pools. The faster C turnover in pools created by ditch blocking than in natural pools suggests that artificial pools are more biogeochemically active than their natural counterparts. Our results, however, point towards a higher proportion and abundance of freshly produced DOM in pools of the natural sites (Figure 3), hence, DOC in natural pools was younger than in most mechanically created pools. As for variations in pool nutrient concentrations, these patterns in DOM composition and concentration may be related to among‐site differences in vegetation cover that influences the production and release of terrestrial, plant‐derived, humic‐like, and protein‐like molecules to the pools (Figure 5; Hassan et al., 2023). Natural pools have high vegetation coverage and thus more stable hydrological conditions, and are likely more biogeochemically active than younger, mechanically created pools.

### Created pools recover natural‐like biogeochemistry over time

Created pool biogeochemistry of eastern Canada varied greatly over time (Table 3), likely due to a combination of water level fluctuations and timing of sampling within the growing season. A recent study of peatland pools showed that DOC concentrations and SUVA in both natural and artificial pools created by ditch‐blocking were higher when pool water level was the shallowest (Chapman et al., 2022). We observed the same trends at our sites, with increasing nutrients and base cations concentrations with decreasing water levels in the created pools. There was, however, no clear pattern of increasing or decreasing pool depth with pool age. Indeed, the hydrology of artificial pools stabilizes only several years after creation in relation to spatially and temporarily variable water table depth in the surrounding peat and differences in plant communities (Holden et al., 2018; McCarter & Price, 2013; Price et al., 2003). While variations in pool depth may drive parts of the biogeochemistry of created pools at different stages of development, peatland hydrology cannot solely explain the differences we observed among the pools we studied.

In peatlands, hydrology and vegetation composition are tightly coupled. For example, differences in water table depth within a single site promote the development of spatially heterogenous plant communities (e.g., Breeuwer et al., 2009). Similarly, vegetation controls water fluxes and creates hydrological gradients because of differential evapotranspiration rates (e.g., Brown et al., 2010). Vegetation in restored peatlands is also related to water table levels that increase over time after the start of restoration efforts (Haapalehto et al., 2011). In restored peatlands, a return to both natural‐like hydrological conditions and vegetation is needed for the reestablishment of C sink functions and biogeochemical cycling (Nugent et al., 2018). The same conditions seem to apply to created pools, with hydrology and vegetation controlling spatial and temporal variations in pool biogeochemistry. The 17‐year post‐creation site is the most similar to natural sites (Figures 5 and 6). This would be explained by higher water table levels at this site compared with the others, leading to more natural hydrological conditions and vegetation around the pools (Figure 1). In the 22 years post‐creation pools, vegetation has successfully recovered (Poulin et al., 2013), but water fluctuation is still important resulting in a pH close to 5, likely preventing the pools from reaching the natural‐like conditions observed at the 17 years post‐creation site. McCarter and Price (2013), who have previously studied the 22 years post‐creation site, suggest that even if a *Sphagnum* cover is present after 10 years, site properties are still distinct from natural peatlands. Accordingly, low *Sphagnum* cover over the peatland does not create a peat layer that is sufficiently thick to retain water and to maintain a high and acidic water table. Hence, pools from older restored sites generally show biogeochemical properties that are more aligned to natural pools than younger created pools, but this is conditional to a return to natural‐like hydrological conditions and vegetation cover.

### Pools in peatland restoration effort and success

The biogeochemistry of natural peatland pools reflects their geography, with spatial and temporal patterns emerging in relation to morphological and biological factors, among others, that constraint water and nutrient cycles. Here, we showed that similar factors control the biogeochemistry of nutrients in created pools, that such cycles differ from those observed in natural pools, and that the differences between natural and created pools tend to diminish over time. Pools that are created in peatland restoration efforts fill much‐needed ecological roles in extracted landscapes (Beadle et al., 2015). Within peatlands, pools are important hydrological buffers (e.g., Arsenault et al., 2019), and biodiversity (Fontaine et al., 2007; Mazerolle et al., 2005) and biogeochemical (e.g., Pelletier et al., 2014) hotspots. In restored peatlands, created pools play similar roles (e.g., Beadle et al., 2023; Holden et al., 2018). Our study further shows that the biogeochemistry of created pools changes over time and would tend to recover nature‐like characteristics after ~20 years conditionally to the reestablishment of stable hydrological conditions and typical peatland vegetation. Based on our results, we postulate that a return of created pools to a natural pool‐like biogeochemistry can inform on the success of peatland restoration.

## CONFLICT OF INTEREST STATEMENT

The authors declare no conflicts of interest.

## Supporting information


Appendix S1.

## Data Availability

Data (Jolin et al., 2024) are available on Zenodo at https://doi.org/10.5281/zenodo.10689626.
