# Supplementary material for: Are pools created when restoring extracted peatlands biogeochemically similar to natural peatland pools?
Source: Ecol Appl. 2024 Oct 11;34(8):e3052. doi: 10.1002/eap.3052 (PMC11610674; doi:10.1002/eap.3052)
Supplement: Supplementary file 1 — Appendix S1. [file EAP-34-e3052-s001.pdf]

Supporting Information. Emilie Jolin, Julien Arsenault, Julie Talbot, Mahmud Hassan, and Line Rochefort. "Are pools created when restoring extracted peatlands biogeochemically similar to natural peatland pools?" Ecological Applications.

Appendix S1.

Table S1. Results of the generalized linear models (GLMs) performed to compare biogeochemical variations among natural created pools in the 2020 and 2021 growing seasons. *Gamma* distribution families were used for all variables. Reference type of pool for each model is in parentheses. Types of pool with different level letters are statistically different at a significance level of 5% ($P < 0.05$).

Response	n	System	Estimate	t	*P*
**Depth**	141	(Created)	19	16.93	-
**(cm)**	130	Natural	75	14.96	<0.001
**Area**	141	(Created)	54	8.09	-
**(m^2)**	130	Natural	324	7.80	<0.001
**DOC**	141	(Created)	99.4	18.64	-
**($mg\ L^{-1}$)**	130	Natural	-67.3	-12.24	<0.001
**pH**	141	(Created)	5.0	146.66	-
	130	Natural	-0.9	-23.02	<0.001
**TN**	141	(Created)	4.6	14.14	-
**($mg\ L^{-1}$)**	130	Natural	-3.7	-11.24	<0.001
**TP**	141	(Created)	120.9	9.82	-
**($\mu g\ L^{-1}$)**	130	Natural	-100.5	-8.09	<0.001
**CO_2**	141	(Created)	3498	8.39	-
**($mg\ L^{-1}$)**	130	Natural	-2189	-5.04	<0.001
**CH_4**	141	(Created)	359.4	5.19	-
**($mg\ L^{-1}$)**	130	Natural	-187.6	-2.54	<0.001
**N_2O**	141	(Created)	309.2	7.09	-
**($mg\ L^{-1}$)**	130	Natural	-307.7	-7.06	<0.001
**SUVA**	141	(Created)	6.04	17.32	-
**($L\ mg\ C^{-1}\ m^{-1}$)**	130	Natural	-2.07	-5.28	<0.001
**NO_3**	141	(Created)	147.3	7.55	-
**($\mu g\ L^{-1}$)**	130	Natural	-139.7	-7.16	<0.001
**NH_4**	141	(Created)	1080.8	6.75	-
**($\mu g\ L^{-1}$)**	130	Natural	-1033.2	-6.44	<0.001
**PO_4**	141	(Created)	22.3	9.23	-
**($\mu g\ L^{-1}$)**	130	Natural	-17.8	-7.28	<0.001
**Ca**	141	(Created)	2.04	12.69	-
**($mg\ L^{-1}$)**	130	Natural	-1.84	-11.38	<0.001
**Mg**	141	(Created)	3.07	12.34	-
**($mg\ L^{-1}$)**	130	Natural	-2.86	-11.48	<0.001
**Na**	141	(Created)	20.64	10.56	-
**($mg\ L^{-1}$)**	130	Natural	-19.36	-9.89	<0.001
**K**	141	(Created)	2.15	10.45	-
**($mg\ L^{-1}$)**	130	Natural	-1.72	-8.22	<0.001

Table S2. Results of the generalized linear models (GLMs) performed to compare biogeochemical variations measured in July 2021 between the natural and the 4, 13, 17, and 22 years post-creation pools. *Gamma* distribution families were used for all variables. Reference type of pool for each model is in parentheses. Types of pool with different level letters are statistically different at a significance level of 5% ($P < 0.05$).

Response	n	System	Estimate	t	*P*	System	Estimate	t	*P*
**Depth (cm)**	9	(Natural)	92	11.95	-	Natural	68	7.68	<0.001
	8	22 years	-76	-9.32	<0.001	22 years	-8	-1.49	0.142
	12	17 years	-37	-3.56	<0.001	17 years	31	3.68	<0.001
	6	13 years	-61	-6.27	<0.001	13 years	8	1.05	0.299
	6	4 years	-68	-7.68	<0.001	(4 years)	24	5.43	-
**Area (m^2)**	9	(Natural)	517	4.46	-	Natural	476	4.03	<0.001
	8	22 years	-469	-3.97	<0.001	22 years	7	0.23	0.822
	12	17 years	-280	-1.96	0.055	17 years	196	2.29	0.026
	6	13 years	-458	-3.82	<0.001	13 years	18	0.51	0.615
	6	4 years	-476	-4.03	<0.001	(4 years)	41	2.02	-
**DOC (mg L^{-1})**	9	(Natural)	30.4	15.16	-	Natural	-123.6	-5.51	<0.001
	8	22 years	68.5	5.45	<0.001	22 years	-54.8	-2.14	0.037
	12	17 years	-5.3	-1.63	0.108	17 years	-128.9	-5.74	<0.001
	6	13 years	31.2	3.41	0.001	13 years	-92.4	-3.84	<0.001
	6	4 years	123.6	5.51	<0.001	(4 years)	154.0	6.90	-
**pH**	9	(Natural)	4.1	74.60	-	Natural	-1.4	-7.99	<0.001
	8	22 years	0.8	5.68	<0.001	22 years	-0.6	-2.90	0.005
	12	17 years	0.3	2.99	0.004	17 years	-1.0	-5.60	<0.001
	6	13 years	1.1	6.89	<0.001	13 years	-0.2	-1.04	0.301
	6	4 years	1.4	8.00	<0.001	(4 years)	5.5	33.93	-
**TN (mg L^{-1})**	9	(Natural)	0.8	23.84	-	Natural	-7.2	-9.75	<0.001
	8	22 years	1.2	7.46	<0.001	22 years	-5.9	-7.88	<0.001
	12	17 years	0.0	0.59	0.556	17 years	-7.2	-9.68	<0.001
	6	13 years	0.2	2.04	0.046	13 years	-7.0	-9.41	<0.001
	6	4 years	7.2	9.75	<0.001	(4 years)	8.0	10.84	-
**TP (µg L^{-1})**	9	(Natural)	12.6	9.16	-	Natural	-71.8	-3.54	<0.001
	8	22 years	29.3	3.33	0.002	22 years	-42.4	-1.93	0.059
	12	17 years	34.2	4.25	<0.001	17 years	-37.6	-1.73	0.089
	6	13 years	41.3	3.18	0.002	13 years	-30.5	-1.27	0.210
	6	4 years	71.8	3.54	<0.001	(4 years)	84.4	4.17	-
**CO_2 (µg L^{-1})**	9	(Natural)	1118	5.93	-	Natural	1115	5.91	<0.001
	8	22 years	617	1.05	0.299	22 years	1732	3.11	0.003
	12	17 years	-421	-1.60	0.114	17 years	693	3.79	<0.001
	6	13 years	1799	1.64	0.117	13 years	2914	2.69	0.009
	6	4 years	-1115	-5.91	<0.001	(4 years)	3	2.69	-
**CH_4 (µg L^{-1})**	9	(Natural)	71.4	4.89	-	Natural	70.9	4.85	<0.001
	8	22 years	-29.2	-1.33	0.190	22 years	41.7	2.53	0.014
	12	17 years	-48.7	-2.99	<0.001	17 years	22.2	3.07	0.003
	6	13 years	109.7	1.33	0.190	13 years	180.6	2.22	0.031
	6	4 years	-70.9	-4.85	<0.001	(4 years)	0.5	2.22	-
**N_2O (µg L^{-1})**	9	(Natural)	3.1	6.25	-	Natural	-1274.6	-2.84	0.006
	8	22 years	-1.7	-2.60	0.12	22 years	-1276.3	-2.84	0.007
	12	17 years	8.1	2.86	0.006	17 years	-1266.5	-2.82	0.007

	6	13 years	23.8	2.51	0.015	13 years	-1250.8	-2.78	0.006
	6	4 years	1274.6	2.84	0.006	(4 years)	1277.7	2.84	-
**SUVA (L mg C^{-1} m^{-1})**	9	(Natural)	4.17	18.73	-	Natural	-0.91	-1.43	0.158
	8	22 years	0.52	1.00	0.322	22 years	-0.39	-0.50	0.616
	12	17 years	-0.02	-0.05	0.961	17 years	-0.93	-1.35	0.182
	6	13 years	0.21	0.37	0.713	13 years	-0.70	-0.90	0.375
	6	4 years	0.91	1.43	0.158	(4 years)	5.08	8.519	-
**NO_3 (µg L^{-1})**	9	(Natural)	9.1	10.45	-	Natural	-112.1	-4.39	<0.001
	8	22 years	46.6	4.57	<0.001	22 years	-65.5	-2.39	0.020
	12	17 years	-0.3	-0.17	0.863	17 years	-112.4	-4.40	<0.001
	6	13 years	13.5	2.79	0.007	13 years	-98.6	-3.80	<0.001
	6	4 years	112.1	4.39	<0.001	(4 years)	121.3	4.75	-
**NH_4 (µg L^{-1})**	9	(Natural)	35.1	7.99	-	Natural	-1543.8	-3.55	<0.001
	8	22 years	58.9	2.58	0.013	22 years	-1484.9	-3.41	0.001
	12	17 years	33.7	2.39	0.020	17 years	-1510.1	-3.48	<0.001
	6	13 years	49.5	2.09	0.041	13 years	-1494.3	-3.44	0.001
	6	4 years	1543.8	3.55	<0.001	(4 years)	1578.9	3.64	-
**PO_4 (µg L^{-1})**	9	(Natural)	3.1	8.40	-	Natural	-17.3	-3.23	0.002
	8	22 years	5.1	2.68	0.010	22 years	-12.2	-2.16	0.035
	12	17 years	4.5	3.09	0.002	17 years	-12.8	-2.32	0.024
	6	13 years	23.0	3.36	0.001	13 years	5.7	0.66	0.512
	6	4 years	17.3	3.23	0.002	(4 years)	20.4	3.82	-
**Ca (mg L^{-1})**	9	(Natural)	0.17	8.05	-	Natural	-1.58	-3.31	0.001
	8	22 years	3.07	4.01	<0.001	22 years	1.49	1.65	0.105
	12	17 years	0.49	3.80	<0.001	17 years	-1.09	-2.21	0.031
	6	13 years	0.43	2.61	0.012	13 years	-1.16	-2.28	0.026
	6	4 years	1.58	3.31	0.002	(4 years)	1.75	3.66	-
**Mg (mg L^{-1})**	9	(Natural)	0.30	9.93	-	Natural	-1.82	-3.88	<0.001
	8	22 years	4.67	4.90	<0.001	22 years	2.85	2.68	0.009
	12	17 years	1.48	5.29	<0.001	17 years	-0.35	-0.64	0.527
	6	13 years	1.26	3.65	<0.001	13 years	-0.56	-0.96	0.341
	6	4 years	1.82	3.88	<0.001	(4 years)	2.12	4.52	-
**Na (mg L^{-1})**	9	(Natural)	1.90	8.26	-	Natural	-14.48	-3.32	0.002
	8	22 years	30.54	4.08	<0.001	22 years	16.05	1.86	0.069
	12	17 years	9.27	4.38	<0.001	17 years	-5.21	-1.08	0.286
	6	13 years	2.30	2.02	0.049	13 years	-12.18	-2.71	0.009
	6	4 years	14.48	3.32	0.002	(4 years)	16.39	3.76	-
**K (mg L^{-1})**	9	(Natural)	0.39	9.75	-	Natural	-3.65	-4.00	<0.001
	8	22 years	0.82	3.42	0.001	22 years	-2.83	-3.01	0.004
	12	17 years	0.56	3.58	<0.001	17 years	-3.09	-3.35	0.001
	6	13 years	0.66	2.76	0.008	13 years	-2.99	-3.17	0.002
	6	4 years	3.65	4.00	<0.001	(4 years)	4.04	4.43	-

Table S3. Summary of generalized linear mixed effects models of water chemistry between natural and created pools, using gamma distribution families. In all models, sites and regions (Québec or New Brunswick) were added as random effects. Asterisks (*) indicate relationships with p-values < 0.05.

Response variable	Explanatory variable	Estimate	Std error	n	t-value	p-value
**DOC ($mg\ L^{-1}$)**	(Created)	87.0	20.9	141	4.167	< 0.001*
	Natural	-59.3	28.5	130	-2.085	0.037*
		Variance of random effects				
	Site	340.60				
	Region	63.85				
	Residuals	0.19				
**pH**	(Created)	5.0	0.2	141	26.22	< 0.001*
	Natural	-0.8	0.4	130	-2.40	0.016*
		Variance of random effects				
	Site	0.02				
	Region	0.00				
	Residuals	0.00				
**TN ($mg\ L^{-1}$)**	(Created)	4.0	1.5	141	2.68	< 0.001*
	Natural	-3.2	2.3	130	-1.35	0.176
		Variance of random effects				
	Site	2.16				
	Region	0.08				
	Residuals	0.24				
**TP ($\mu g\ L^{-1}$)**	(Created)	90.2	19.9	141	4.53	< 0.001*
	Natural	-75.9	28.0	130	-2.71	0.007*
		Variance of random effects				
	Site	994.56				
	Region	157.35				
	Residuals	0.74				
**CH_4 ($\mu g\ L^{-1}$)**	(Created)	359.4	33.4	141	10.76	< 0.001*
	Natural	-187.6	35.5	130	-5.28	< 0.001*
		Variance of random effects				
	Site	0.00				
	Region	0.00				
	Residuals	3.76				
**N_2O ($\mu g\ L^{-1}$)**	(Created)	312.0	39.3	141	7.93	< 0.001*
	Natural	-308.4	39.3	130	-7.85	< 0.001*
		Variance of random effects				
	Site	0.00				
	Region	10.48				
	Residuals	1.57				
**NO_3 ($\mu g\ L^{-1}$)**	(Created)	147.3	13.6	141	10.81	< 0.001*
	Natural	-139.7	13.6	130	-10.25	< 0.001*
		Variance of random effects				
	Site	0.00				
	Region	0.00				
	Residuals	1.78				
**NH_4**	(Created)	1080.8	71.0	141	15.22	< 0.001*

**(μg L^{-1})**	Natural	-1033.2	71.1	130	-14.53	< 0.001*
		Variance of random effects				
	Site	0.00				
	Region	0.00				
	Residuals	2.23				
**PO_4 (μg L^{-1})**	(Created)	20.7	4.0	141	5.19	< 0.001*
	Natural	-16.6	5.9	130	-2.85	0.004*
		Variance of random effects				
	Site	58.91				
	Region	0.00				
	Residuals	1.07				
**SUVA (L mg C^{-1} m^{-1})**	(Created)	5.4	0.7	141	8.17	< 0.001*
	Natural	-1.8	0.7	130	-2.34	0.019*
		Variance of random effects				
	Site	0.38				
	Region	0.20				
	Residuals	0.32				
**Ca (mg L^{-1})**	(Created)	1.53	0.43	141	3.58	< 0.001*
	Natural	-1.44	0.51	130	-2.84	0.005*
		Variance of random effects				
	Site	0.17				
	Region	0.06				
	Residuals	0.41				
**Mg (mg L^{-1})**	(Created)	2.53	0.49	141	5.20	< 0.001*
	Natural	-2.21	0.86	130	-2.58	0.010*
		Variance of random effects				
	Site	0.31				
	Region	0.00				
	Residuals	0.21				
**Na (mg L^{-1})**	(Created)	17.22	4.20	141	4.10	< 0.001*
	Natural	-14.95	7.22	130	-2.07	0.038*
		Variance of random effects				
	Site	1.74				
	Region	0.00				
	Residuals	0.16				
**K (mg L^{-1})**	(Created)	1.96	0.54	141	3.61	< 0.001*
	Natural	-1.50	0.85	130	-1.76	0.078
		Variance of random effects				
	Site	0.49				
	Region	0.00				
	Residuals	0.37				

Table S4. Summary of generalized linear mixed effects models of pH, DOC, TN and TP variations over the 2020 and 2021 growing seasons at the Québec sites, using gamma distribution families. In all models, pool number and sampling time were added as random effects. Asterisks (*) indicate relationships with p-values < 0.05.

**Response variable**	**Explanatory variable**	**Estimate**	**Std error**	**n**	**t-value**	**p-value**
**DOC ($mg\ L^{-1}$)**	(22 years)	85.2	5.5	64	15.533	< 0.001*
	4 years	65.4	11.3	48	5.788	< 0.001*
	Natural	-50.9	5.9	160	-8.581	< 0.001*
		Variance of random effects				
	Pool number	44.68				
	Sampling time	7.26				
	Residuals	0.14				
**pH**	(22 years)	4.9	0.1	64	54.779	< 0.001*
	4 years	0.4	0.1	48	3.290	0.001*
	Natural	-0.8	0.1	160	-8.084	< 0.001*
		Variance of random effects				
	Pool number	0.01				
	Sampling time	0.00				
	Residuals	0.00				
**TN ($mg\ L^{-1}$)**	(22 years)	2.8	0.2	64	15.830	< 0.001*
	4 years	6.1	0.6	48	10.220	< 0.001*
	Natural	-1.8	0.2	160	-10.980	< 0.001*
		Variance of random effects				
	Pool number	0.02				
	Sampling time	0.02				
	Residuals	0.18				
**TP ($\mu g\ L^{-1}$)**	(22 years)	169.5	14.0	64	12.131	< 0.001*
	4 years	-91.1	16.0	48	-5.708	< 0.001*
	Natural	-148.1	14.0	160	-10.611	< 0.001*
		Variance of random effects				
	Pool number	15.01				
	Sampling time	27.34				
	Residuals	0.59				

Table S5. Spectral characteristics of the three components validated by PARAFAC modeling, number of matches in *OpenFluor* (minimum similarity of 0.95).

Component	Maximum excitation (nm)	Maximum emission (nm)	Number of *OpenFluor matches*	Sources
C1	250	440	80	Terrestrially derived humic-like DOM with high molecular weight degraded from lignin
C2	250	516	54	Terrestrial humic-like DOM
C3	345	470	4	Terrestrial humic-like, high relative aromaticity and molecular weight
C4	270	326	76	Protein-like (mixture of tyrosine-type and tryptophane-type compounds), autochthonous DOM

Table S6. Means of physical and chemical properties of natural and created pools from Québec with standard deviations for eight-sample campaign over two growing seasons (2020-2021) where the precision of mean values is related to the accuracy of the instruments used for the analyses.

**Pools Properties**	**Natural QC**	**22 Years**	**3 Years**
Number of individual pools	21	8	6
Number of pools sampling	164	50	34
Depth (cm)	93 (±50)	17 (± 3)	24 (± 2)
Area (m^2)	347 (± 509)	49 (± 12)	40 (± 6)
Underlying peat (cm)	407 (±50)	126 (±11)	42 (±6)
pH	4.1 (± 0.2)	4.9 (± 0.3)	5.3 (± 0.2)
DOC (mg L^{-1})	32.4 (± 10.3)	85.6 (± 39.1)	152.7 (± 102.2)
TN (mg L^{-1})	0.9 (± 0.3)	2.8 (± 1.8)	9.0 (± 5.8)
TP (µg L^{-1})	21.1 (± 13.2)	176.1 (± 234.3)	77.8 (± 54.0)
NH_4 (µg L^{-1})	48.2 (± 57.6)	263.4 (± 857.7)	2815.9 (± 2672.6)
NO_3 (µg L^{-1})	7.6 (± 4.7)	64.8 (± 171.6)	339.3 (± 411.6)
PO_4 (µg L^{-1})	4.6 (± 4.5)	17.3 (± 25.6)	34.3 (± 31.1)
SUVA (L mg C^{-1} m^{-1})	3.99 (± 0.92)	5.71 (± 4.70)	7.48 (± 7.30)
CH_4-C (µg L^{-1})	180 (± 321)	705 (± 962)	134 (± 320)
CO_2-C (µg L^{-1})	1339 (± 1068)	6408 (± 7017	974 (± 616)
N_2O-N (µg L^{-1})	1.2 (± 0.8)	1.5 (± 1.0)	3.9 (± 4.7)
Ca (mg L^{-1})	0.21 (± 0.11)	3.12 (± 1.50)	1.68 (± 1.38)
Mg (mg L^{-1})	0.17 (± 0.09)	4.49 (± 1.46)	2.25 (± 1.76)
K (mg L^{-1})	0.43 (± 0.26)	1.28 (± 0.57)	4.29 (± 2.66)
Na (mg L^{-1})	0.91 (±0.45)	30.65 (± 11.96)	16.62 (± 11.99)
